# Full and Partial Agonists of Thromboxane Prostanoid Receptor Unveil Fine Tuning of Receptor Superactive Conformation and G Protein Activation

**DOI:** 10.1371/journal.pone.0060475

**Published:** 2013-03-28

**Authors:** Valérie Capra, Marta Busnelli, Alessandro Perenna, Manuela Ambrosio, Maria Rosa Accomazzo, Celine Galés, Bice Chini, G. Enrico Rovati

**Affiliations:** 1 Department of Pharmacological and Biomolecular Sciences, Università degli Studi di Milano, Milan, Italy; 2 CNR Institute of Neuroscience, Milan, Italy; 3 Department of Medical Biotechnology and Translational Medicine, Università degli Studi di Milano, Milan, Italy; 4 Institute of Pharmacology and Toxicology, University of Würzburg, Würzburg, Germany; 5 Institute des Maladies Métaboliques et Cardiovasculaires, INSERM, U1048, Université Toulouse III Paul Sabatier, Centre Hospitalier Universitaire de Toulouse, Toulouse, France; University of São Paulo, Brazil

## Abstract

The intrahelical salt bridge between E/D^3.49^ and R^3.50^ within the E/DRY motif on helix 3 (H3) and the interhelical hydrogen bonding between the E/DRY and residues on H6 are thought to be critical in stabilizing the class A G protein-coupled receptors in their inactive state. Removal of these interactions is expected to generate constitutively active receptors. This study examines how neutralization of E^3.49/6.30^ in the thromboxane prostanoid (TP) receptor alters ligand binding, basal, and agonist-induced activity and investigates the molecular mechanisms of G protein activation. We demonstrate here that a panel of full and partial agonists showed an increase in affinity and potency for E129V and E240V mutants. Yet, even augmenting the sensitivity to detect constitutive activity (CA) with overexpression of the receptor or the G protein revealed resistance to an increase in basal activity, while retaining fully the ability to cause agonist-induced signaling. However, direct G protein activation measured through bioluminescence resonance energy transfer (BRET) indicates that these mutants more efficiently communicate and/or activate their cognate G proteins. These results suggest the existence of additional constrains governing the shift of TP receptor to its active state, together with an increase propensity of these mutants to agonist-induced signaling, corroborating their definition as superactive mutants. The particular nature of the TP receptor as somehow “resistant” to CA should be examined in the context of its pathophysiological role in the cardiovascular system. Evolutionary forces may have favored regulation mechanisms leading to low basal activity and selected against more highly active phenotypes.

## Introduction

The prostanoid receptor for thromboxane A_2_ (TXA_2_), called TP, belongs to the Class A (rhodopsin family) of the superfamily of heptahelical transmembrane receptors, commonly referred to as G protein-coupled receptors (GPCRs), the most diverse form of transmembrane signaling protein and the most privileged target of marketed drugs. The TP receptor was originally purified from human platelets and successively cloned from human placenta [1]. The G protein-coupling repertoire for TP receptors is rather extensive. It is classically considered a Gq-coupled receptor activating the PLCβ – IP_3_/DAG – Ca^++^/PKC signaling cascade, based on the phylogenetic and experimental analysis [2], [3]. However, it has been shown to couple also to G_s_, G_i_ and G_12/13_ [1]. In humans, TP receptor exists in two isoforms sharing the first 328 amino acids, TPα (343 residues) and TPβ (407 residues), which is an alternative mRNA splicing variant with an extended carboxyl terminus.

The TXA_2_/TP receptor system is of great pathophysiological importance in the cardiovascular system. Indeed, TP receptor activation produces platelet shape change and aggregation, providing a positive stimulus for causing thrombus formation. Furthermore, the equilibrium between platelet-derived TXA_2_ and endothelial-derived prostacyclin represents the rationale for the use of anti-thrombotic low-dose aspirin, but also the proposed cause of cardiovascular side effects of COX-2 selective inhibitors [4]. TP receptor expression and activity account for its involvement in diseases based on endothelial dysfunction and proliferation such as atherosclerosis [5], and cancer [6]. In this context, TP receptor function appears to be tightly regulated at gene and protein level. Accordingly, the deleterious cardiovascular effects of TPα could be limited by heterodimerization with the alternatively spliced TPβ [7], [8] or the counteracting prostacyclin receptor IP [9], [10], which have been shown to regulate its trafficking and G protein coupling.

Many issues regarding GPCR function are still unclear despite a number of (seventeen) GPCRs have been crystallized so far, from rhodopsin to ß-adrenergic (ARs), muscarinic, and more recently opioid receptors. A common feature thought to be important in the process of activation of many class A GPCRs is the network of interactions carried out between the charged R^3.50^ in the conserved E/DRY motif at the end of helix 3 (H3) and the E^6.30^ in H6, the so called cytoplasmic ionic lock, and the E/D^3.49^ in the intrahelical salt bridge. This network of interactions is observed in all of the inactive rhodopsin crystal structures [11], in the dopamine D_3_ receptor [12] and in a limited subset of A_2A_ [13] and β_1_-AR [14] structures, and has been implicated through mutagenesis as a major factor stabilizing receptors in their inactive conformation [15], [16].

We previously showed that neutralization of R^3.50^ in the TP receptor did not result in a constitutively active mutant (CAM), but assigned Arg a dual role in participating in the reinforced hydrogen bond network of the ionic lock and in direct binding with the G protein [15], [17]. As suggested by molecular dynamic (MD) simulations of TP receptor [18], we formerly observed that neutralization of E^3.49^ and E^6.30^ resulted in mutants characterized by a maximum U46619 response larger than in wild-type (WT). However, these mutants lacked any elevation of basal G-protein/effector activity, a phenotype clearly different from the constitutively active that we named superactive [19]. This contribution is aimed to demonstrate that the observed phenotype is not a feature restricted to a single agonist, but rather a fundamental characteristic of the superactive mutants (SAMs), and that other hallmarks of the active-like conformation are preserved defining a unique pharmacological profile for these proteins. Crucial points were to challenge the peculiar resistance of TP to a ligand-independent activity and to gain information on the molecular mechanism underlying increased agonist-induced activation and signaling (superactivity).

## Materials and Methods

### Reagents

Cell-culture media, animal serum, antibiotics, other supplements, Lipofectamine 2000, Opti-MEM I and molecular biology reagents were purchased from Invitrogen (Carlsbad, CA). Inositol-free Dulbecco's modified Eagle's medium (DMEM) was obtained from ICN Pharmaceuticals Inc. (Costa Mesa, CA). Ultima Gold was from PerkinElmer Life and Analytical Sciences (Boston, MA), as were [5,6-^3^H]SQ29,548 and myo-[2-^3^H]inositol. U46619 ([1R-[1α,4α,5β(Z),6α(1E,3S*)]]-7-[6-(3-hydroxy-1-octenyl)-2-oxabicyclo[2.2.1]hept-5-yl]- 5-heptenoic acid), I-BOP ([1S-[1α,2α(Z),3β(1E,3S*),4α]]-7-[3-[3-hydroxy-4-(4-iodophenoxy)-1-butenyl]-7-oxabicyclo[2.2.1]hept-2-yl]-5-heptenoic acid), 8-isoPGF_2α_ (8-iso Prostaglandin F_2α_), 8-isoPGE_2_ (8-iso Prostaglandin E_2_), PTA_2_ (Pinane TXA_2_ - 9α,11α-(dimethyl)methylene-15S-hydroxy-11α-deoxy-11α-methylene-thromba-5Z,13E-dien-1-oic acid) and SQ29,548 ([1S-[1α,2α(Z),3α,4α]]-7-[3-[[2-[(phenylamino)carbonyl]-hydrazino]methyl]-7-oxabicyclo[2.2.1]hept-2-yl]-5-heptenoic acid) were from Cayman Chemical (Ann Arbor, MI). Coelenterazine 400a (CLz400) and coelenterazine h were from Biotium (Hayward, CA). Anion exchange resin AG 1X-8 (formate form, 200–400 mesh) and Lowry dye-binding protein reagents were from Bio-Rad (Hercules, CA). All other reagents of the highest purity were available from Sigma-Aldrich (St. Louis, MO).

### Constructs

DNA constructs of TP receptor WT, E129V, and E240V were previously obtained in our laboratory [17], [19]. E129V/E240V substitutions were introduced into the cDNA for human TPα receptor using the same mutant oligonucleotides and method used to obtain the single mutant receptors, as previously published [19] and the identity of the double mutant was assessed by sequencing. The cDNA encoding for the Gαq was purchased at Missouri S&T cDNA Research Center (Rolla, MO, USA). The plasmids encoding GFP^10^-Gγ2 and Gβ1 have been previously described [20] and the expression vector for Gq proteins fused to *Renilla* luciferase, that brings eight favor mutations (Gαq-Rluc8) cDNA is described in Saulière et al., [21]. Ultrapure plasmids for cell transfection were obtained using the QIAfilter Plasmid Kits by Qiagen (Hilden, Germany).

### Cell culture and transient transfections

HEK293 transfection host cells were purchased from the American Type Culture Collection (ATCC, Manassas, VA). Cells were routinely grown in Dulbecco's modified Eagle's medium (DMEM) supplemented with 10% FBS, 2 mM glutamine, 100 U/ml penicillin, 100 µg/ml streptomycin and 20 mM HEPES buffer pH 7.4, at 37°C in a humidified atmosphere of 95% air and 5% CO_2_. For transfection, cells were seeded onto tissue culture dishes previously coated with 10 µg/ml poly-D-lysine, and transfected at 50–60% confluence with an optimized 2∶1 Lipofectamine 2000/DNA ratio following manufacturer's instructions, as described previously [19]. All assays were performed 48 hours after transfection. In cotransfection experiments with TP and Gαq, plasmids were added in a 1∶3 and 1∶5 µg ratio, respectively.

### Radioligand binding and total inositol phosphate assays

Receptor expression and functional activity were monitored 48 hours after transfection. Ligand binding characteristics were determined on confluent adherent cells performing a mixed-type protocol [22] with the specific receptor antagonist [^3^H]SQ29,548 (48 Ci/mmol) as previously described [17], [19]. Heterologous competition studies involved concentrations of the indicated unlabeled ligands extending from 0.1 nM to 30 µM. After 30 min incubation at 25 °C, the reaction was stopped by aspiration of the medium, cells were washed with ice-cold PBS containing 0.2% (w/v) BSA and lysed in 0.5 N NaOH. Radioactivity was measured by liquid scintillation counting. Binding data were analyzed as described in Data and Statistical Analysis. Quantification of the total labeled inositol phosphates (IP) accumulation was performed using a conventional gravity flow column chromatography, as described previously [17], [19]. Briefly, the day before the assay, cells were labeled with 0,5 µCi of [myo-2-^3^H]inositol (17 Ci/mmol) for 18–20 hours in serum-free, inositol-free DMEM containing 20 mM HEPES buffer, pH 7.4, and 0.5% (w/v) Albumax I. The day of the assay, media was replaced with serum-free, inositol-free DMEM containing 25 mM LiCl and cells stimulated for 30 min with the indicated agonists. After removal of the medium, cells were lysed with 10 mM formic acid and lysates were applied onto an anion exchange AG 1X-8 column, formate form, 200–400 mesh. The total IP fraction was then eluted with 2 M ammonium formate/formic acid buffer at pH 5 and radioactivity determined by liquid scintillation counting. Rescue experiments were performed essentially as above, except for a 18 hours incubation step with 1 µΜof SQ29,584 followed by its removal by washing three times with ice-cold PBS containing 1 mM MgCl_2_ and 0.1 mM CaCl_2_ before starting the experiment.

### Bioluminescent resonance energy transfer

HEK 293 cells were co-transfected with vectors expressing the GFP^10^-Gγ_2_, Gβ_1_, Gα_q_-Rluc8 and WT or E129V mutant of TP receptor to investigate direct G protein activation with an intramolecular bioluminescent resonance energy transfer (BRET^2^) strategy. To avoid possible variations in the BRET signaling resulting from fluctuation in the relative expression levels of the energy donor and acceptor, we designed transfection conditions to maintain constant GFP^10^ and Rluc8 expression and their ratios in each experimental set. Total fluorescence and luminescence were directly determined in an aliquot of the transfected cells using an Infinite F500 microplate reader (Tecan, Milan, Italy) as previously described [23]. WT and mutant TP receptor level of expression, evaluated by binding experiments, were comparable in each experimental set and constant among experiments. 48 hours after transfection, cells were washed once with PBS, detached, and resuspended in PBS+0.1% (w/v) glucose at room temperature. Cells were then distributed (80 µg of proteins per well) into a 96-well microplate (Wallac, Perkin Elmer, Monza, Italy) and incubated in the presence or absence of increasing concentrations of ligands for 2 min before the addition of the luciferase substrate Coelenterazine 400a (5 µM; Biotium, Hayward, CA). BRET signal between Rluc8 and GFP^10^ was measured immediately after the addition of the Rluc substrate in the microplate reader. The BRET signal was calculated as the ratio of the light emitted by GFP^10^ (510–540 nm) over the light emitted by Rluc8 (370–450 nm). The changes in BRET induced by the ligands were expressed as Òagonist-induced BRETÓ, obtained by subtracting the BRET signal detected in the presence of PBS by the BRET signal detected in the presence of the specific ligand.

### Data and statistical analysis

All average results are presented as mean±S.E. When indicated, ANOVA followed by post-hoc test for multiple comparisons was performed. Data from radioligand binding were evaluated by a nonlinear, least-squares curve-fitting procedure using GraphPad Prism version 4, implemented with the n-ligand m-binding site model, as described in the LIGAND computer program [24]. Concentration-response curves were evaluated using Prism 4, which use the four parameters logistic model as described in the ALLFIT program [25]. Parameter errors are all expressed in percentage coefficient of variation (%CV) and calculated by simultaneous analysis of at least two different independent experiments performed in duplicate or triplicate. Parameter comparison has been performed on the base of the F test for extra sum of square principle. All curves shown are computer generated.

## Results

### Analysis of Wild Type and Mutant TP Receptors Expression

Binding assays were performed with the specific antagonist [^3^H]SQ29,548 in HEK293 cells transiently transfected with WT, mutants of the human TPα receptor isoform, or vector alone. Mock-transfected cells showed no binding to [^3^H]SQ29,548 (data not shown). WT and mutant receptors were expressed at a level sufficient to perform radioligand binding analysis, yet, to allow a proper comparison of receptor response. Thus, transfection conditions were adjusted to secure equivalent levels of receptor expression for WT and mutants as previously described [8], [17], [19]. Computer-assisted analysis of binding data from WT receptor and mutants revealed, as expected for neutral antagonists (see below), monophasic binding curves fitting a single site model. Calculated affinities for the WT and mutant receptors were in the nanomolar range, as previously reported [17], [19], while capacities ranged approximately from 0.3 to 0.9 pmol/mg protein, a level comparable to receptor expression in human platelets (Table 1). All the experiments were conducted within this range of receptor expression, if not specified differently.

**Table 1:** Binding affinities of [^3^H]SQ29,548 in HEK293 cells transiently expressing the WT or mutant human TP receptors.

Receptor	Gαq^a^	K_d_, nM±%CV	B_max_, pmol/mg prot±%CV
WT	-	11.25±20	0.44±32
WT	3x	15.50±11	0.90±46
WT	5x	8.85±12	0.95±39
E129V	-	9.25±19	0.53±16
E129V	3x	8.45±29	0.38±21
E129V	5x	7.98±28	0.39±30
E240V	-	10.03±15	0.97±31
E240V	3x	7.53±33	0.65±52
E240V	5x	8.50±53	0.49±34
E129/240V	-	9.06±52^b^	0.04±68 b

Binding affinities and capacities were obtained by simultaneous analysis of at least 3 independent mixed-type experiments each performed in duplicates, analyzed with GraphPad Prism implemented with the LIGAND model (see Data and statistical analysis).

a In cotransfection experiments, TP and Gαq plasmids were added in a 1∶3 (3x) and 1∶5 (5x) ratio (see methods for details).

b Parameters refer to E129/240V double mutant before SQ29,548 rescue.

### Agonist-induced signaling and binding profile

**Figure 1:**
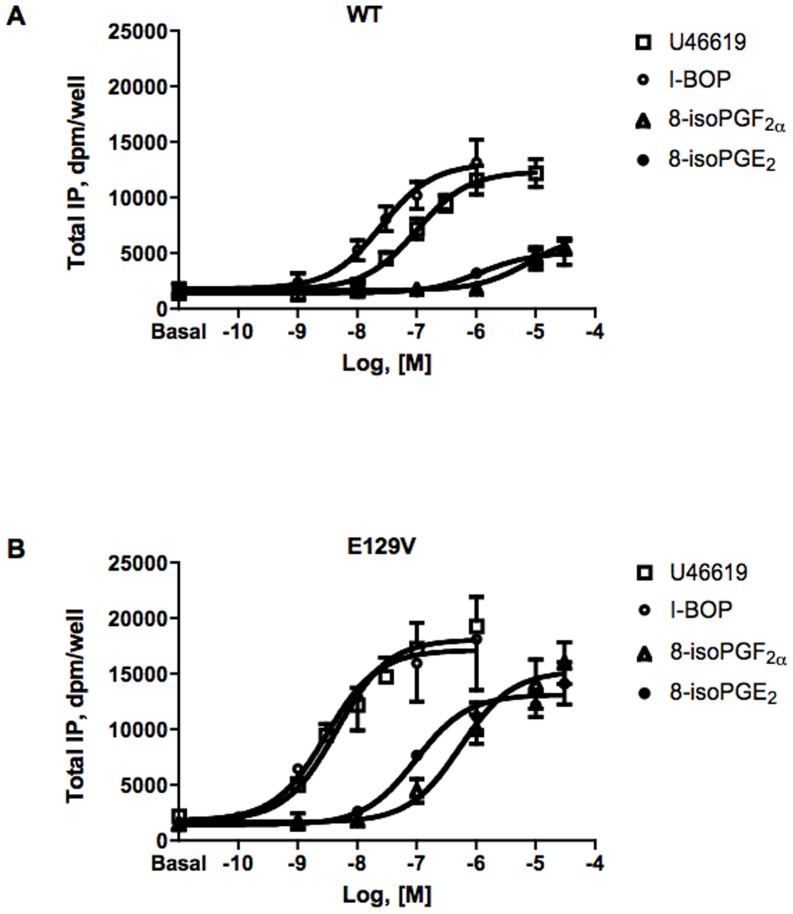
Agonist-induced total IP accumulation in HEK293 cells transiently expressing equal amounts of the WT (A) or E129V mutant (B) of human TP receptor. Total IP accumulation was measured after incubation in the absence (basal) or presence of increasing concentrations of the indicated agonists for 30 min. Data are expressed as dpm/well. Error bars represent mean±SE of at least three independent experiments each performed in duplicates or triplicates (For the sake of clarity, in panel B, error bar direction of U46619 and I-BOP data is above and below, respectively). Curves are computer generated from the simultaneous analysis of at least three independent experiments. Values for EC_50_ and significant differences from WT are shown in Table 2.

**Figure 2:**
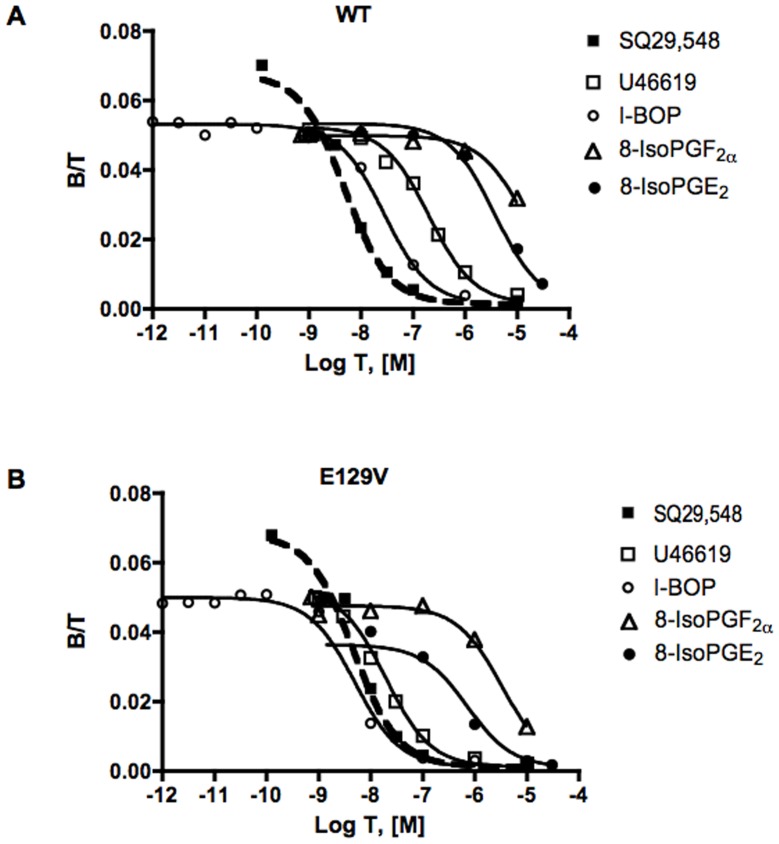
Agonist binding studies in HEK293 cells transiently expressing the WT (A) or E129V mutant (B) of human TP receptor. For each construct, cold U46619, I-BOP, 8-isoPGF_2α_ or 8-isoPGE_2_ was used in competition for 1 nM [^3^H]SQ29,548. Mixed type curves and heterologous competition curves were performed at 25°C with 30 min incubation. Binding is expressed as the ratio of bound ligand concentration to total ligand concentration (B/T, dimensionless) versus the logarithm of total unlabeled ligand concentration (Log T). Non-specific binding was calculated by computer as one of the unknown parameters of the model and was always <10% of total binding. Curves are computer generated from the simultaneous analysis of several independent mixed-type and heterologous competition experiments, each in duplicate. Values for K_i_ and significant differences from WT are shown in Table 3.

We previously observed that the TXA_2_ stable analog U46619 stimulated total IP formation by E129V and E240V mutants with a significant 10-fold lower EC_50_ and higher E_max_ than the WT receptor [19]. To further expand these observations and assess if these specific stimulation characteristics are also be found for other agonists, providing stronger evidence for superactivity, agonist-induced concentration-response curves were obtained for a series of TP agonists, such as the full agonist I-BOP and the partial agonists isoprostanes, 8-iso-PGF_2α_ and 8-isoPGE_2_ in E129V, assumed as a standard example of SAM. Computer assisted analysis showed that the EC_50_s of the agonists for E129V were significantly 10 to 20 fold leftward shifted (p<0.01) with respect to that of the WT (Figure 1 and Table 2). In addition, the full agonist I-BOP showed a greater efficacy in activating the E129V SAM compared to the WT TP receptor in agreement with data from U46619, whereas the intrinsic activity of the two partial agonists 8-iso-PGF_2α_ and 8-isoPGE_2_ was augmented, with maximum efficacies not statistically different from those of the full agonists (Figure 1B and Table 2). We also previously demonstrated that the heterologous competition curves of the unlabeled agonist U46619 vs. [^3^H]-SQ29,548 revealed a leftward shift for the E129V mutant curve compared to WT receptor [19]. Thus, binding of the panel of agonists was performed to assess whether the mutation modified their binding profile. Figure 2 clearly shows that the entire panel of agonists, including the two partial agonists, reveals a significant (p<0.01) increase in affinity up to one order of magnitude (Table 3), confirming a unique binding profile for the E129V SAM with respect to different structural and pharmacodynamic classes of compounds. These data, therefore, suggest a change in the receptor conformation, as identified by an increase in agonist affinities, but similar conserved folding and quaternary structure, as identified by unchanged antagonist affinity.

**Table 2:** Total IP dose-response parameters for different agonists in HEK293 cells transiently expressing the WT or the E129V TP receptor.

	WT		E129V		
Agonist	EC_50_, nM±%CV	E_max_, dpm/well±%CV	EC_50_, nM±%CV	E_max_, dpm/wel±%CV	Activity ratio^a^
U46619	98.68±70	12374±8	4.47±36**	18115±5	22.1
I-BOP	25.11±65	13099±14	2.99±56**	17156±8	8.4
8-isoPGE_2_	1155±72	5087±21	95.93±60**	12201±5	12.0
8-isoPGF_2α_	3999±68	6744±42	493.1±53**	15287±7	8.1

Values of EC_50_ and E_max_ were obtained by simultaneous analysis with GraphPad Prism (see Data and statistical analysis) of at least three independent experiments each performed in duplicates.

a Activity ratio was calculated as the ratio of the EC_50_ for the WT TP receptor over the EC_50_ of the E129V mutant.

** p<0.01 vs. WT.

**Table 3:** Affinities of the agonists for the binding site of the receptor labelled by [^3^H]SQ29,548 in HEK293 cells transiently expressing the WT or the mutant human TP receptors.

Agonist	WT K_i_, nM±%CV	E129V K_i_, nM±%CV	Affinity ratio^a^
U46619	169.90±13	16.32±18**	10.4
I-BOP	24.27±18	3.90±21**	6.2
8-isoPGE_2_	2916±15	547.6±2**	5.3
8-isoPGF_2α_	12930±19	2908±11**	4.4

Ki values were obtained by simultaneous analysis of at least 3 independent competition experiments analyzed with GraphPad Prism implemented with the LIGAND model (see Data and statistical analysis).

a Affinity ratio was calculated as the ratio of the K_i_ for the WT TP receptor over the K_i_ of the E129V mutant.

** p<0.01 vs. WT.

### Analysis of Basal Activity and U46619-induced response in the presence of overexpression of Gαq

**Figure 3:**
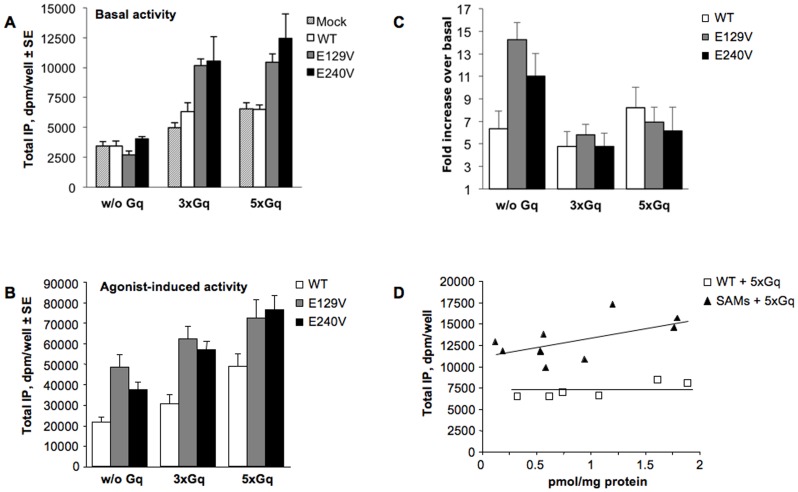
Analysis of basal and agonist induced total IP accumulation in HEK293 cells transiently expressing the WT or SAM mutants of human TP receptor in the absence and presence of Gαq overexpression. TP and Gαq plasmids were added in a 1∶3 (3x) and 1∶5 ratio (5x), respectively. A. Total IP accumulation in basal conditions. B. Total IP accumulation induced by 30 min stimulation with 1 µM U46619. C. Fold increase over basal of total IP accumulation. D. Basal activity for WT and SAMs with increasing receptor expression in pmol/mg protein. Data are expressed as dpm/well. Error bars represent mean±SE of at least three independent experiments each performed in duplicates or triplicates.

A key feature that we previously observed in SAMs of TP receptor is that, albeit adopting an active-like conformation, they lack the increase in basal activity [15], [19]. Since theoretically it can be anticipated that the raise in G protein concentration should shift the R-R* equilibrium to favor the formation of the active R*, the basal activities of WT and SAM TP receptors were assayed by analyzing their ability to activate production of total IP in the absence of agonist stimulation, but in the presence of overexpression of Gαq. Here we confirm that, in the absence of Gq overexpression none of the TP receptor mutants exhibited CA when expressed at equal protein level (Fig. 3A, w/o Gq). In addition, considering that the solely overexpression of Gq in the absence of any TP receptor expression (mock, textured bars) induced a dose-dependent increase of total IP production (43% and 118% for -3 and 5-fold Gq respectively, Fig. 3A), we can conclude that the enhance in IP accumulation is not driven by WT TP receptor expression (white vs. textured bars). E129V and E240V yielded only a modest 2-fold increase in agonist-independent activity (Figure 3A, solid gray and black bars vs. textured bars). Interestingly, U46619-induced total IP production is amplified in the presence of increasing amount of Gq protein (Figure 3B) and its potency is augmented (data not shown), as it would be expected by an increased availability of signaling proteins. Yet, in the latter condition the agonist-induced activation of SAM receptors showed a fold-increase in total IP production that is comparable to the WT (Figure 3C). This behavior is not typical for CAM receptors, which are usually strongly impaired in agonist-induced stimulation as their basal activity increase and get close to the maximal response allowed by the system [26], [27]. Performing transfections with increasing DNA concentrations to augment receptor expression in conditions of Gαq overexpression (to avoid G protein depletion) there was a slight but not significant increase in basal activity for the SAM with respect to the WT receptor (Figure 3D).

As predicted by the ternary complex model, the affinity for the labeled [^3^H]-SQ29,548 did not show any variation between WT and SAMs in the presence of overexpression of Gαq with respect to basal G protein expression (see Table 1).

### Pharmacological characterization of the E129V/E240V double mutant

**Figure 4:**
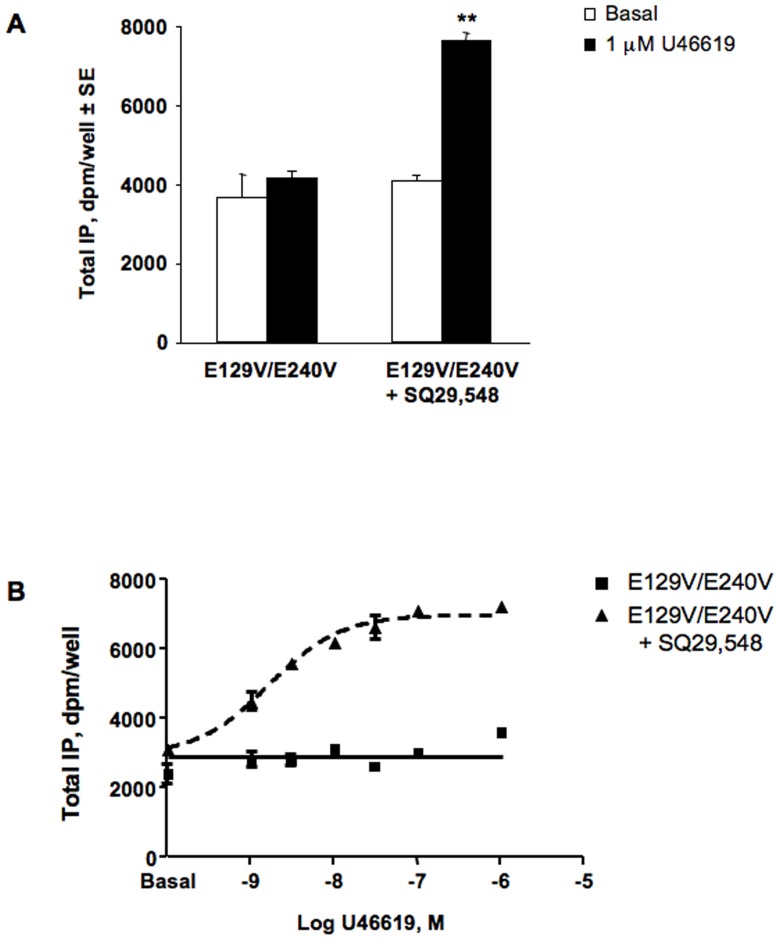
Functional analysis of basal and agonist induced total IP accumulation in HEK293 cells transiently expressing the double mutant E129V/E240V of human TP receptor without and following rescue with 1 µ**M SQ29,548 for 18** **h.** A. Total IP accumulation in basal condition and following 30 min stimulation with 1 µM U46619. B. Concentration-response curves of U46619. Error bars represent mean±SE of at least two independent experiments each performed in duplicates.

To further characterize the behavior of a receptor bearing multiple mutations possibly affecting its conformation in a broader and unexplored way, we constructed the E129V/E240V double mutant. Expression levels of E129V/E240V were negligible making impossible to perform significant analysis of functional activity, despite similar antagonist affinity to WT (Table 1). We therefore explored the possibility that the double mutant might be retained and not sufficiently available for signal transduction by performing functional rescue experiments exposing cells to 1 µM SQ29,548 for 18 hours [28]. Analysis of agonist-induced signal transduction was performed after removal of SQ29,548 by repeating washing (see methods). Figure 4A clearly shows that SQ29,548 is able to rescue double mutant receptor functional activity, albeit not completely compared to the WT or single mutant phenotypes (see Figure 1). The general features of this mutant are consistent with those observed for each single SAM. Analysis of basal activity and of the concentration-response curve of U46619 revealed the absence of CA (compare with Figure 3A) and an EC_50_ value of 1.66 nM±33%CV consistent with those previously obtained for SAMs [19].

### Pharmacodynamic analysis of TP antagonists in the presence of Gαq overexpression

**Figure 5:**
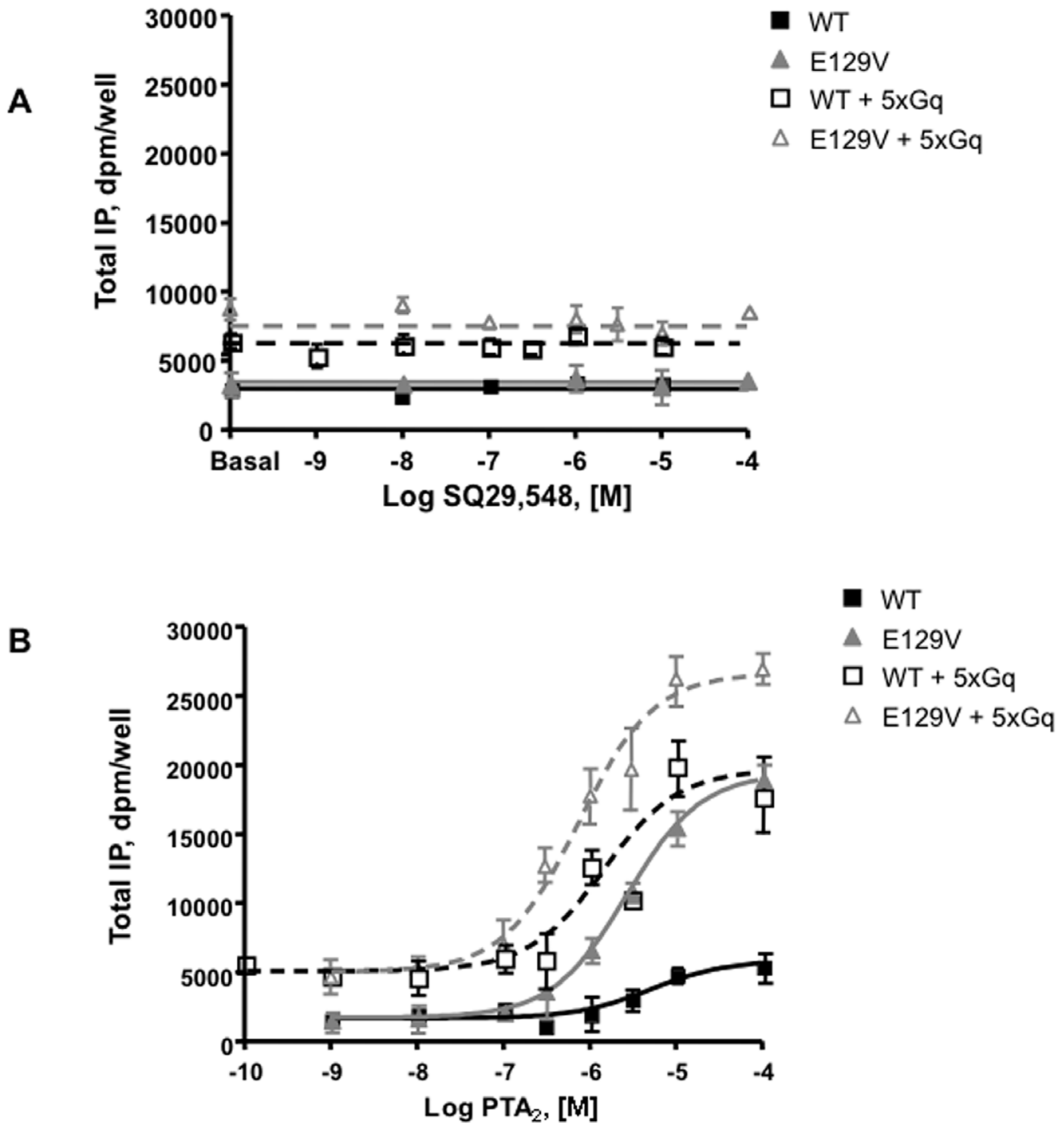
Pharmacodynamic analysis of antagonists SQ29,548 and PTA_2_ in functional assay. Total IP accumulation in HEK293 cells transiently expressing the WT or E129V mutant of human TP receptor was assayed in the absence and presence of Gαq overexpression at increasing concentrations of the antagonists for 30 min. TP and Gαq plasmids were added in a 1∶5 (5x) ratio. Data are expressed as dpm/well. Error bars represent mean±SE of at least three independent experiments each performed in duplicates or triplicates. Curves are computer generated from the simultaneous analysis of several independent experiments.

SQ29,548 behaved as a pure antagonist in either the absence or presence of Gαq overexpression with respect to WT and E129V mutant (Figure 5A). Of interest, the purported antagonist pinane-TXA_2_ (PTA_2_) [29] behaved as a very weak partial agonist with respect to WT TP receptor (E_max_ 5856 dpm/well±14%CV), but as a much stronger agonist with respect to E129V SAM (E_max_ 19423 dpm/well±2%CV) as well as in conditions of Gαq overexpression (EC_50_ 1447 nM±67%CV and E_max_ 19670 dpm/well±15%CV for WT; EC_50_ 747 nM±76%CV; E_max_ 26642 dpm/well±5%CV for E129V) (Figure 5B). Similar K_i_ has also been obtained in heterologous competition experiments for PTA_2_ between WT and E129V (Ki 12140 nM±39%CV and 4836 nM±31%CV, respectively). Overall, these observations suggest that the SAM has the propensity to assume a conformation more prone to be activated by a weak agonist, but despite the availability of an excess of signaling molecules, i.e. Gαq proteins in this case, they are “resistant” to CA.

### Direct G protein activation

**Figure 6:**
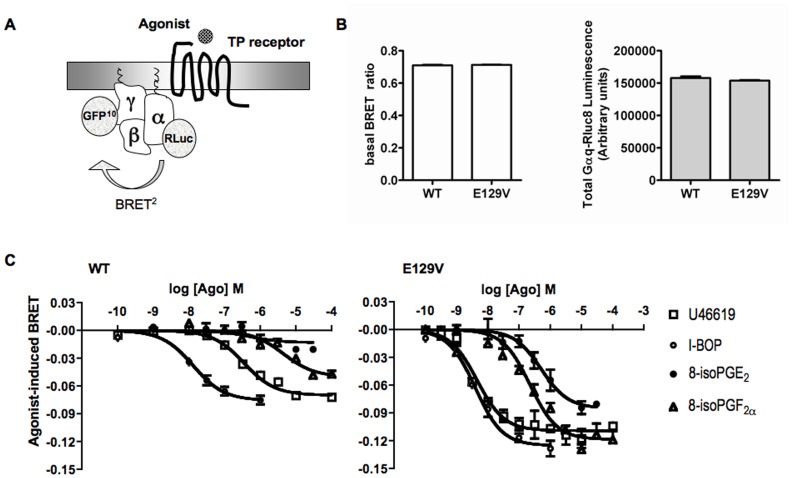
BRET^2^ measurement of Gα_q_β_1_γ_2_ complex activation in HEK293 living cells expressing equal amounts of the WT of human TP receptor or its E129V mutant. A. BRET^2^ was measured between the donor Rluc8 and the acceptor GFP^10^ introduced at the residue 97 of the Gα_q_ subunit and the N-terminal domain of the Gγ_2_ subunit, respectively. Agonist-induced coupling of TP receptor and Gq protein distances Gα_q_-Rluc8 and GFP^10^-Gγ_2_ giving rise to a decrease in the BRET signal. B. Protein expression levels of the constructs used for BRET experiments were set to be constant and able to assure the same level of basal BRET ratio in the presence of WT and E129V mutant of the human TP receptors. Total Gα_q_-Rluc8 luminescence was evaluated in HEK293 cells co-expressing Gα_q_-Rluc8 together with GFP^10^-Gγ_2_ and Gβ_1_ in the presence of WT or E129V mutant of the human TP receptor measuring the light emission in aliquots of the transfected cells incubated with 5 µM coelenterazine for 8 min. In the same cells stimulated with PBS, basal BRET ratio was calculated as the ratio of the light emitted by GFP^10^ (510–540 nm) over the light emitted by Rluc8 (370–450 nm). C. BRET was measured in HEK293 cells co-expressing Gα_q_-Rluc8 together with GFP^10^-Gγ_2_ and Gβ_1_ in the presence of WT (left) or E129V (right) mutant of the human TP receptor and stimulated with increasing concentrations of the indicated full and partial agonists. Results are the differences in the BRET signal measured in the presence and the absence of agonists, and are expressed as the mean value±SE of at least two independent determinations.

To challenge predictions from MD simulation suggesting a more “efficient” coupling of SAMs with their cognate G protein compared to WT receptor [19], we performed a direct measure of G protein activation by using an intramolecular BRET^2^ strategy in which Renilla reniformis luciferase 8 (Rluc8) [30] is the energy donor and GFP^10^ is the energy acceptor. This recorded real time conformational changes between the α and the βγ subunits within the Gq protein, where a decrease of the BRET signal provoked by agonist-induced receptor activation is indicative of a conformational reorganization leading the Gα-Gβγ interface to open, reflects the initial event of Gq protein activation [20], [23] (Figure 6A).

After setting the optimal transfection conditions (Figure 6B) to avoid possible variations in the BRET signal resulting from fluctuations in the relative expression levels of the energy donor, evaluated as the total Gαq-Rluc8 luciferase emission, and acceptor and to assure the same level of basal BRET signal for WT and SAMs (1∶1∶1.25 α∶β∶γ ratio), concentration-response curves of the stable agonist U46619, I-BOP and of the isoprostanes 8-iso-PGF_2α_ and 8-isoPGE_2_ have been obtained in HEK293 cells expressing equal amount of WT and E129V receptors. As it is clear from Figure 6C, U46619 and I-BOP curves obtained with the E129V mutant show an increased efficacy and more than one order of magnitude leftward shift with respect to the curves obtained with the WT TP receptor. Similarly, isoprostanes show an increase in potency and intrinsic activity when activating the E129V mutant. Analysis of the data indicated EC_50_ values for E129V and WT that are in good agreement with those obtained from the analysis of total IP dose-response curves (compare Table 2 and 4). Statistical comparison of potencies and efficacies provided a significant difference between parameters obtained with WT and E129V, thus demonstrating a more “efficient” G protein activation by E129V mutant protein (Table 4).

**Table 4:** BRET concentration-response parameters for different agonists in HEK293 cells transiently expressing the WT or the E129V TP receptor.

	WT		E129V		
Agonist	EC50, nM±%CV	Emax,±%CV	EC50, nM±%CV	Emax, dpm/well±%CV	Activity ratioa
U46619	345.4±14	−0.07±3	4.53±25**	−0.11±4	76
I-BOP	13.01±27	−0.07±5	3.96±22**	−0.12±4	3.3
8-isoPGE2	1983±72	−0.02±42	484.8±46*	−0.08±8	4.1
8-isoPGF2α	3936±48	−0.05±9	404.1±31**	−0.12±4	9.7

Values of EC_50_ and E_max_ were obtained by simultaneous analysis with GraphPad Prism (see Data and statistical analysis) of at least two independent experiments each performed in duplicates.

a Activity ratio was calculated as the ratio of the EC_50_ for the WT TP receptor over the EC_50_ of the E129V mutant.

* p<0.05, ** p<0.01 vs. WT.

## Discussion

In many GPCRs, the intrahelical salt bridge between E/D^3.49^ and R^3.50^ within the E/DRY motif on H3 and the interhelical hydrogen bonding between the R^3.50^ and residues on H6 are thought to form a network that is disrupted during receptor activation, unlashing constraints imposed on the two helices [31]. These data have been more recently confirmed by several crystal structures of activated opsin [32], [33] or constitutively active rhodopsin [34], [35]. Mutations on both sides of this network were shown to increase basal activity of a number of receptors, including another prostanoid receptor, FP [36], yet not in all [15].

The extended ternary complex model (ETC) of interactions among receptor, ligand, and G protein was first suggested by pharmacological analysis of adrenoreceptor CAMs [37]. Several features were predicted based on this model that make a distinction between CAMs and WT receptors. These characteristics include an increase in ligand-independent activity, an increase in ligand affinity/potency that correlates with compound pharmacodynamic (i.e., largest shifts for full agonists, no changes for antagonists), and a systematic amplification of partial agonist efficacy.

We previously observed that introduction of valine, one of the most non-conservative mutation affecting the extent of CA [26], at the E^3.49^ and E^6.30^ produced TP mutants characterized by enhanced agonist potency/efficacy upon activation by the TXA_2_ stable analog U46619 with respect to both Gq and Gs coupling [19]. These traits are indicative of a mutation-induced active-like conformation, which however did not trigger ligand-independent signaling in contrast with the ETC prediction. For this distinctive phenotype, these mutants were named superactive mutants (SAMs) in alternative to CAMs [19], as has been very recently observed also for the bradykinin B_2_ receptor [38].

The current study examines how SAMs of the TP receptor alter ligand-induced binding and signaling with respect to WT. Here we demonstrate that the increase in agonist potency is not a feature of a single agonist (U46619), but is shared by a panel of full and partial agonists. This observation is important to rule out the possibility that the effect observed might be ligand-specific [39], but to emphasize an intrinsic characteristic of the mutant protein. Furthermore, the partial agonist isoprostanes displayed an increased intrinsic activity, behaving as full agonists, whereas the purported antagonist PTA_2_ behaved as partial agonist. Interestingly, also the affinities of all the agonists were increased in ligand-binding studies performed with the E129V mutant. All together, these results strongly indicate that these mutated proteins adopt a particular conformation indicative of a superactive state, which is recognized by different full and partial agonists, and that identifies a unique pharmacological profile. Of notice, the unaltered binding characteristics of the neutral antagonist SQ29,548 suggest that the folding and the overall conformation of SAM was not grossly perturbed by the mutations. As expected, antagonist potencies calculated in functional studies are only apparently reduced, due to an increase in agonist potency.

The sensitivity with which CA is detected is influenced by the level of expression of receptor and G protein and the relative stoichiometry to each other. Therefore, we studied whether rising receptor or Gαq protein levels might boost ligand-independent activity of WT and SAMs. Our data clearly demonstrate that basal activity of WT TP receptor is neither affected by amplification of receptor expression, nor of its cognate G protein. A modest increase in CA of E129V and E240V SAMs can be observed only after a substantial overexpression of Gαq, but not of Gαq and receptor. While this behavior is rather predictable due to the presence of an increased availability of signaling molecules (as demonstrated by the increase in basal IP production in cells transfected solely with Gαq), it really seems of little physiological importance considering that both SAMs retain fully their ability to cause agonist-induced signaling, something largely impaired in a CAM. Thus, we have separated two fundamental aspects of receptor function, basal and agonist-induced activity, supporting the notion that the active receptor conformation induced by agonists might be substantially different from that caused by constitutive activation [39], [40].

To test the hypothesis that CA might has gone unrecognized in SAMs bearing a single amino acid mutation, we have also generated and tested the double mutant E129V/E240V. Our data reveal that without rescued cell surface expression of TP double mutant, the cells lack the ability to generate IP in response to U46619, indicating that the double mutant protein is somewhat misrouted. However, restoring receptor to function again did not reveal any increase in basal activity, yet showed potency similar to that of E129V and E240V single SAMs. Therefore, we can conclude that TP receptor basal activity is not or only marginally affected by disruption of the extended network involving the conserved ERY motif.

Of notice, despite the presence of residues capable of forming the interhelical ionic lock, this interaction has not been found in the majority of the GPCR crystal structures resolved so far (regardless if they have been obtained in the presence of antagonists or inverse agonists), with the exception of rhodopsin [11], D_3_ receptors [12] and, to a limited subset of antagonist-bound A_2A_ [13] and β_1_-AR [14] structures. Nevertheless, this interaction may still exist in the native receptors, as long time scale MD simulations performed on the β_2_-AR [41], [42], β_1_-AR [43] and A_2A_R [44] show that the ionic lock reforms and breaks, suggesting existence of equilibrium states characterized by the presence and the absence of the lock that might reflect different requirements for a basal activity in a physiological context. Therefore, receptors might simply have a different propensity to form this interhelical contact in the contest of the conformational plasticity characteristic of GPCRs, which might explain the agonist-independent activation observed for some of them. Interestingly, rhodopsin [45], D_3_ [46] as well as the TP receptor have no or very low basal CA, at variance with what has been reported for the β_2_-AR (even bound to the inverse agonist carazolol [47]), β_1_-AR and A_2A_ [27], [48], [49].

In addition, Vogel and colleagues have demonstrated that neutralization of the intrahelical salt bridge of rhodopsin is considerably more critical for shifting the photoproduct equilibrium to an active-like conformation than the disruption of the interhelical bonding between R^3.50^ and E^6.30^ [16]. This salt bridge is indeed present in rhodopsin [11], D_3_ [12] and β_2_-AR [47], [50] bound to an antagonist or inverse agonist, and broken in both opsin [32] and β_2_-AR [51] active structures. However, while mutational studies on the E/D^3.49^ of opsin [45], [52] or D_3_ receptor [46] indicate only a partial or no increase in basal activity, the same mutation in the β_2_-AR clearly produce a CAM [53]. Because our data indicate that neither disruption of the intrahelical, nor of the interhelical (or both) interaction(s) affect TP basal activity, we might conclude that disruption of this network might be necessary but not sufficient to achieve full receptor activation. Indeed, the crystallographic structures of opsin [33] or even β_2_-AR [54] in their G protein-interacting conformation show significant rearrangement of the transmembrane regions beyond the disruption of the entire E/DRY network, suggesting that its neutralization might be only one of multiple constraints that must be “unlashed” to achieve full receptor activation. Indeed, similar behavior has been observed for other GPCRs [15], thus it seems conceivable that some receptors have developed a large activation energy barrier, while others might have a low energy barrier between the R and R* states leading, in the latter case, to a relevant basal activity [55].

We might speculate here that the increased affinity for full and partial agonists demonstrated by our SAMs might be due to an allosteric effect of the mutations at the agonist binding site, independent of G protein coupling. Alternatively, the mutation might shift the equilibrium toward one of the receptor species theorized by the Cubic Ternary Complex model of Weiss and colleagues, characterized by an increased ability to bind, but not activate G proteins [56]. Indeed, the release of R^3.50^ from the wrapped conformation in the ground state might allow the binding of the Gα protein in a position previously occupied by H6 [32], [33], [34], [35]. These findings are consistent with the loss of agonist-induced signaling/G protein coupling observed upon mutation of R^3.50^ for TP [17] as well as for a number of other class A GPCRs [15], including rhodopsin [45], [57] and dopamine D_3_ receptor [46], just to limit to those that have been crystallized. Furthermore, MD simulations of TP SAMs in complex with heterotrimeric G_q_ revealed a better predisposition of R^3.50^ to perform connections at the receptor-G protein interface, as demonstrated by a larger solvent accessible surface (SAS) area in the cytosolic regions of the E129V mutant, compared to the WT [19]. Thus, the effects of E^3.49^ mutation on the structural features of TP is suggestive of an increased efficiency in G protein coupling. This prediction has been challenged *in vitro* by performing a BRET^2^-based assay to measure early activation of Gq protein reflecting events close to the receptor and therefore not subjected to signaling amplification, feedback and cross regulation from different effector systems that may complicate interpretation of the results. Monitoring BRET signal variations resulting from structural rearrangements within the heterotrimeric Gαq and βγ protein in real time in living cells, we observed a significant leftward shift of the BRET concentration-response curves (augmented potency) and a larger decrease in BRET signal (augmented efficacy) in the E129V dose-response curves for all the agonists tested. In addition, for both the full agonist U46619 and the partial agonist 8-iso-PGF_2α_ the leftward shift between WT and E129V mutant is larger in BRET signal (potency ratio of 76 and 9.7, respectively. See Table 4) than in binding affinities (K_i_ ratio of 10 and 4.4, respectively. See Table 3), between WT and E129V mutant. Overall, these observations may be interpreted as a magnification of the “efficiency” with which SAM directly promotes agonist-induced G protein activation. This, in turn, can be the result of an increased receptor-G protein interaction due to an enhanced G protein affinity for the receptor, or, alternatively, to an increase in the rate of GDP/GTP exchange. In either case, this effect should be ascribed to a change in the receptor conformation of the SAMs.

## Conclusions

Collectively, pharmacological data lead us to show not only that non-conservative substitution of E^3.49^ and E^6.30^ TP results in mutants characterized by higher affinity/potency for different agonists, and increased intrinsic activity for partial agonists, but also that SAMs are more efficient in G protein coupling and activation. However, the low intrinsic basal activity of the TP relative to that of other GPCRs, indicates that the equilibrium between R and R* in TP strongly favors the inactive form. The failure of TP to be constitutively activated upon mutations in the hydrogen bond network indicates that its disruption is not the only structural feature required to activate the G protein, but rather that additional mechanism(s) govern the R-R* transition, playing variable roles in different GPCRs. In this light, and considering the TXA_2_/TP receptor relevance in the pathophysiology of the cardiovascular system, evolutionary forces may have favored regulatory mechanisms leading to low basal activity and selected against more highly active phenotypes.
